# From El Niño forecasts to proportionate public health action: a forecast-to-action framework for climate-related health risks

**DOI:** 10.3389/fpubh.2026.1971395

**Published:** 2026-09-23

**Authors:** Leena H. Bajrai, Esam I. Azhar

**Affiliations:** 1 Department of Biochemistry, Faculty of Sciences, King Abdulaziz University, Jeddah, Saudi Arabia; 2 Special Infectious Agents Unit–BSL-3, King Fahd Medical Research Center, King Abdulaziz University, Jeddah, Saudi Arabia; 3 Department of Medical Laboratory Sciences, Faculty of Applied Medical Sciences, King Abdulaziz University, Jeddah, Saudi Arabia

**Keywords:** anticipatory action, climate variability, climate-sensitive diseases, early warning, El Niño, ENSO, health equity, health-system resilience

## Abstract

El Niño creates an important opportunity for anticipatory public health action because its development can often be detected before many downstream hazards emerge. Yet predictability does not transfer uniformly from the large-scale El Niño Southern Oscillation (ENSO) signal to regional climate anomalies and ultimately to health outcomes. This Perspective proposes a locally adaptable forecast-to-action framework that distinguishes three levels of predictability: ENSO prediction, regional and local climate prediction, and health-risk prediction. We argue that an El Niño forecast should initiate a structured risk-assessment process rather than automatically trigger intervention. Decisions should integrate forecast probability and skill, expected hazard severity, local epidemiology and vulnerability, lead time, intervention effectiveness and reversibility, equity, and the consequences of acting versus not acting. Low-cost, no-regret measures can reasonably begin at lower levels of certainty, whereas costly or disruptive interventions require stronger evidence. Because empirical evidence for forecast-triggered interventions remains limited, prospective implementation and evaluation are essential. Linking climate information with local surveillance, One Health intelligence, resilient health systems, equity-sensitive decision-making, and continuous learning may convert forecast lead time into earlier, proportionate, and more defensible public health action.

## 1 Introduction

El Niño is one of the major sources of interannual climate variability. Through anomalous warming of the central and eastern tropical Pacific Ocean and accompanying changes in atmospheric circulation, it can influence temperature and precipitation across widely separated regions. Its effects, however, are not geographically uniform: drought may dominate in one setting, heavy rainfall or flooding in another, while some regions experience heat extremes or comparatively modest effects (1–3).

The public health importance of this variability extends well beyond meteorology. Climate influences heat exposure, water availability, food production, vector ecology, air quality, population mobility, and the conditions under which healthcare is delivered. El Niño-related health concerns can therefore include heat-related illness, vector- and waterborne infections, food insecurity and malnutrition, respiratory and cardiovascular morbidity, displacement, psychosocial stress, and interruption of essential health services (2–4).

The issue is particularly timely. In July 2026, the World Meteorological Organization reported that a strong El Niño was developing and expected to intensify during August–October 2026, while emphasizing that seasonal temperature and rainfall outlooks remain probabilistic and geographically variable (1). WHO has similarly highlighted the value of anticipatory preparedness for heat-related illness, vector- and waterborne diseases, malnutrition, and disruption of essential health services (4).

These warnings provide valuable lead time, but they also highlight an important limitation. Predicting El Niño is not equivalent to predicting a local health emergency. A reliable ENSO forecast may improve understanding of future climate risk, but whether that signal translates into a local environmental hazard—and whether the hazard subsequently produces disease or health-system disruption—depends on teleconnections, baseline epidemiology, infrastructure, population vulnerability, adaptive capacity, and interventions already in place.

Earlier perspectives appropriately called for stronger ENSO-related health preparedness and for multisectoral responses adapted to local circumstances (2, 3). The incremental contribution of this Perspective is to focus on the next step: how probabilistic climate information can be translated into proportionate, locally adaptable, and evaluable public health decisions.

We propose that an ENSO forecast should begin a structured forecast-to-action process rather than automatically activate a predetermined intervention. The central task is to determine when available evidence from climate prediction, local environmental conditions, epidemiological surveillance, population vulnerability, health-system capacity, and intervention characteristics is sufficient to justify action—and what level of action is proportionate to that evidence.

## 2 From ENSO prediction to health-risk prediction: three levels of certainty

The pathway from climate forecast to health outcome contains at least three distinct levels of predictability. Keeping them separate is essential for responsible decision-making.

### 2.1 ENSO prediction

The first level is prediction of the ENSO state itself. Contemporary dynamical and statistical systems can provide useful probabilistic forecasts of El Niño, La Niña, or neutral conditions several months in advance. However, real-time evaluations demonstrate that ENSO forecast skill varies according to lead time, forecast initialization, model characteristics, season, and the event being predicted (5).

An ENSO forecast should therefore be interpreted as a probabilistic estimate of the future state of the tropical Pacific Ocean atmosphere system. It is not, by itself, a prediction of drought, flooding, dengue, malnutrition, heat-related illness, or hospital demand in a specific population.

### 2.2 Regional and local climate prediction

The second level concerns how the ENSO signal translates into regional or local temperature, rainfall, drought, flooding, or other environmental hazards. These relationships depend on atmospheric teleconnections whose strength and direction vary geographically and seasonally. Other modes of climate variability and local physical conditions may amplify, weaken, or alter the ENSO signal.

This distinction is especially relevant to seasonal decision-making. Recent work from the Arabian Peninsula, for example, demonstrates meaningful ENSO-related predictability of boreal summer temperatures in some areas while also showing substantial spatial variation in forecast performance (6). A high-confidence ENSO forecast can therefore coexist with considerably lower confidence about the environmental conditions that will occur in a particular locality.

### 2.3 Health-risk prediction

The third level—prediction of health outcomes—is more conditional still. Even when a regional climate anomaly is forecast correctly, its health consequences depend on baseline disease epidemiology, population immunity, vector and reservoir ecology, exposure, housing and sanitation, socioeconomic conditions, health-seeking behavior, healthcare access, and the effectiveness of ongoing interventions (7).

Dengue illustrates this complexity. Recent multicountry analyses strengthen evidence that ENSO variability can influence dengue risk, but they also demonstrate substantial geographic heterogeneity associated with local temperature and precipitation teleconnections (8, 9). ENSO-related variation in diarrheal disease is similarly context dependent. In Botswana, La Niña rather than El Niño was associated with increased childhood diarrhea under particular environmental circumstances, whereas recent Chinese surveillance demonstrates geographically heterogeneous ENSO–diarrheal disease relationships (10, 11).

These findings are not contradictory. They demonstrate why a global climate signal must be interpreted through local environmental, epidemiological, and social conditions.

Predictability therefore generally becomes more conditional along the pathway:

ENSO state → regional/local climate hazard → local health outcome.

Recognizing this decline in certainty should not delay preparedness. Rather, it should ensure that the intensity, cost, and potential disruption of the response remain proportionate to the strength and local relevance of the available evidence.

The proposed framework is summarized in Figure 1.

**Figure 1:**
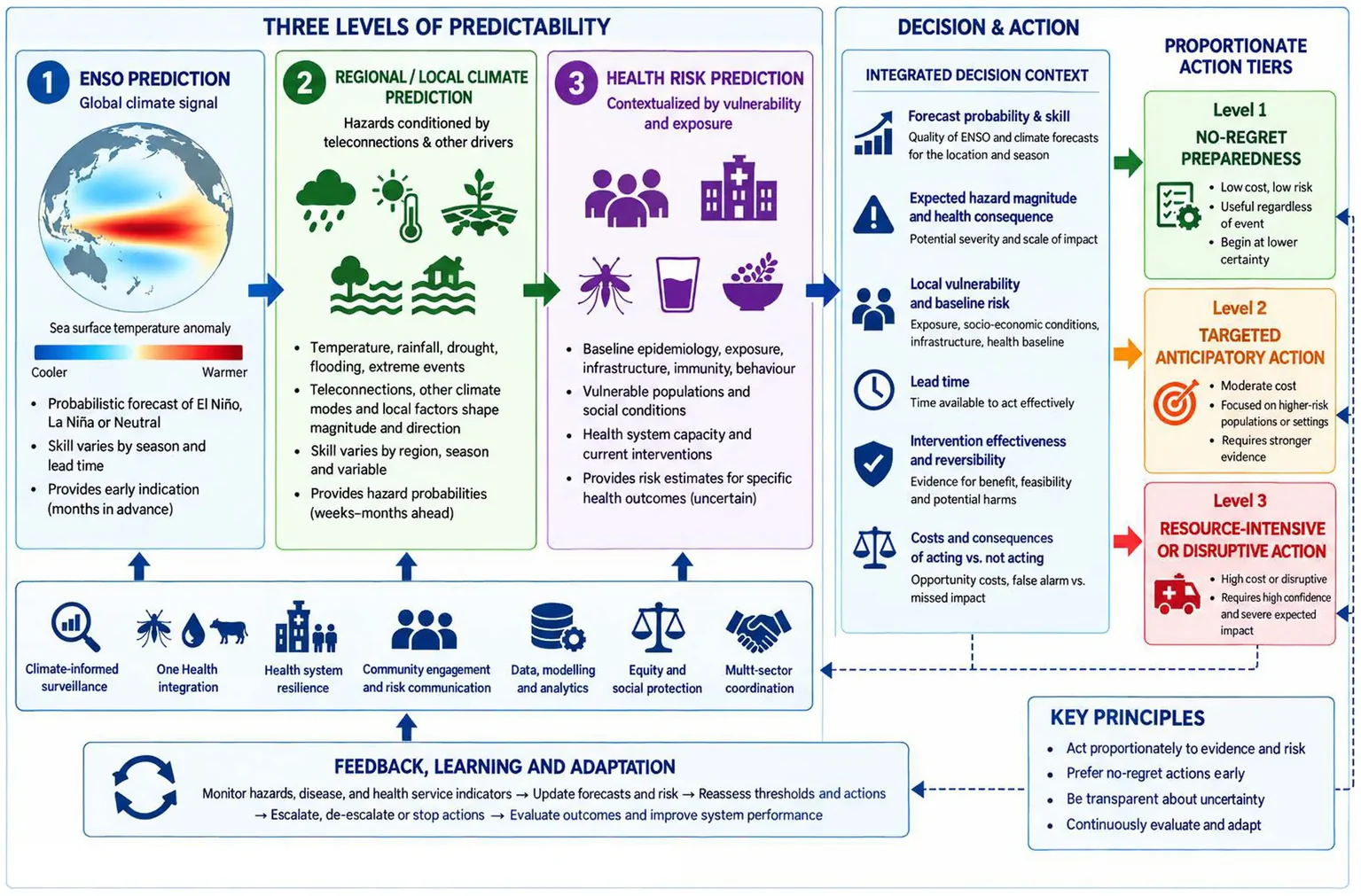
Forecast-to-action framework for proportionate and locally adaptable El Niño-related public health preparedness. The framework distinguishes three successive levels of predictability: ENSO prediction, regional/local climate prediction, and health-risk prediction. Downstream risk becomes increasingly dependent on teleconnections, baseline epidemiology, exposure, population vulnerability, infrastructure, health-system capacity, and ongoing interventions. These inputs feed an integrated decision process incorporating forecast probability and skill, expected hazard severity, local vulnerability and baseline risk, available lead time, intervention effectiveness and reversibility, equity, and the costs and consequences of acting versus not acting. Actions are graduated from low-cost/no-regret preparedness to targeted anticipatory measures and, when evidence is sufficiently strong, resource-intensive or disruptive interventions. Thresholds should be calibrated to local climate, epidemiology, health-system capacity, resources, and social context. Continuous surveillance, One Health integration, multisectoral coordination, community engagement, and evaluation provide feedback for escalation, de-escalation, recalibration, and learning.

## 3 From forecast to locally adapted and proportionate action

The concept of a forecast “trigger” is useful only if the trigger is clearly defined. In this framework, El Niño itself is not the trigger. A trigger is a locally agreed decision rule indicating that available evidence has reached a level at which a specified action is justified.

WHO guidance on climate-informed early-warning systems emphasizes the need to connect environmental monitoring and forecasting with active surveillance, communication, mechanisms for early response, and assessment of operational usefulness (12). A recent systematic review of climate-informed early-warning systems for vector-borne diseases further highlights persistent challenges related to fragmented data, model validation, forecast interpretation, institutional coordination, and translation of forecasts into timely action (13).

Experience from anticipatory humanitarian action offers complementary lessons. Forecast-triggered systems are most useful when event probability is considered alongside expected impact, population vulnerability, lead time, and the costs of acting unnecessarily versus failing to act (14, 15).

For public health, seven considerations should inform the decision threshold.

### 3.1 Forecast probability and skill

A high probability estimate has limited value if the forecasting system has poor historical performance for the relevant location, season, or hazard. Probability should therefore be interpreted alongside demonstrated forecast skill.

### 3.2 Expected hazard severity and health consequences

The level of certainty required for action should partly reflect what is at stake. A severe but plausible threat affecting a highly vulnerable population may justify earlier preparedness than a relatively minor expected impact.

### 3.3 Local vulnerability and baseline risk

Identical climate forecasts may appropriately produce different decisions in different settings because populations differ in disease burden, age structure, occupational exposure, housing, water security, healthcare access, and health-system resilience.

### 3.4 Lead time

An intervention is useful only when the warning arrives early enough for meaningful implementation. Longer lead times may create greater opportunities for preparedness but may also involve greater forecast uncertainty.

### 3.5 Intervention effectiveness and reversibility

Measures that are safe, inexpensive, broadly beneficial, and readily reversible can reasonably be initiated with less certainty than expensive, disruptive, or difficult-to-reverse interventions.

### 3.6 Consequences of action and inaction

False alarms may consume resources and reduce confidence, whereas missed warnings may result in preventable illness, deaths, or disruption of essential services.

### 3.7 Equity and distribution of benefit and burden

A decision should consider not only the average expected health benefit but also who receives protection, who bears the costs of intervention, and whether an action could widen existing inequalities. A technically optimal intervention may still be inappropriate if it systematically excludes populations with the greatest vulnerability or least capacity to respond.

This reasoning supports three broad tiers of action.

### 3.8 No-regret preparedness

Low-cost measures with value even if the predicted event does not occur can begin relatively early. Examples include reviewing surveillance trends, checking laboratory and diagnostic readiness, verifying essential stocks, updating clinical guidance, identifying vulnerable populations, assessing continuity plans, and strengthening communication between meteorological and public health authorities.

### 3.9 Targeted anticipatory action

As local climate forecasts strengthen, or epidemiological, environmental, and vulnerability indicators point toward increasing risk, more focused measures may be justified. These could include intensified entomological surveillance, enhanced water-quality monitoring, targeted heat-health communication, additional nutritional surveillance, or proactive outreach to high-risk communities.

### 3.10 Resource-intensive or disruptive action

Measures that carry substantial financial, logistical, or social costs should generally require stronger and more locally specific evidence. The expected health impact should be meaningful, the intervention feasible and likely to provide benefit, and the consequences of acting unnecessarily explicitly considered. Where feasible, predefined review points should allow interventions to be escalated, de-escalated, modified, or stopped as new information becomes available.

### 3.11 Local adaptation and calibration

A central feature of the framework is that the structure can be standardized, but the thresholds should not be.

Countries and subnational jurisdictions should adapt the framework using their own climate history, forecast performance, disease epidemiology, social vulnerability, health-system capacity, resource constraints, and institutional arrangements. For example, a heat-alert threshold appropriate for a highly acclimatized population with widespread cooling access may be unsuitable for a population where housing, electricity access, or baseline cardiovascular risk differs substantially.

Similarly, an ENSO-linked dengue preparedness trigger should reflect whether the area has established *Aedes* populations, historical climate–dengue associations, population susceptibility, diagnostic capacity, and effective vector-control options.

Local adaptation could therefore include:

- Retrospective assessment of forecast skill and historical climate–health relationships;
- Identification of locally important hazards and vulnerable populations;
- Selection of measurable environmental, epidemiological, and health-service indicators;
- Locally agreed trigger thresholds;
- Specification of actions linked to each threshold;
- Assessment of feasibility, resource requirements, and equity implications; and
- Predefined intervals for reassessment and recalibration.

The framework should therefore function as a decision architecture, not as a universal algorithm.

## 4 Evidence for risk is not the same as evidence for action

A further distinction is critical when translating climate science into practice. Evidence that a climate exposure is associated with a health outcome does not necessarily establish that a proposed intervention will be effective when activated because of an ENSO forecast.

The evidence base is uneven. For some hazards, climate–health relationships are comparatively well established and effective public health interventions are available. What is often missing is direct evidence that activating these interventions earlier on the basis of seasonal climate information produces additional health benefit. Table 1 therefore separates the evidence supporting the climate health relationship from the evidence supporting the proposed intervention.

**Table 1:** El Niño-associated health pathways, anticipatory options, local adaptation, and the evidence supporting action.

Hazard or pathway	Evidence for climate–health relationship	Anticipatory action and local adaptation	Evidence supporting intervention and remaining gaps
Extreme heat	High temperatures are strongly associated with heat-related illness and worsening cardiovascular, renal, and respiratory disease (2–4)	Heat-health warnings, occupational protection, cooling measures, clinical readiness, and targeted outreach. Thresholds should reflect local acclimatization, housing, electricity access, age structure, and occupational exposure	Heat-health warning systems have a growing evidence base, but effectiveness varies with thresholds, communication, infrastructure, and population response. Evidence on the incremental benefit of ENSO-informed activation remains limited (17)
Heavy rainfall, flooding, and unsafe water	Flooding, sanitation disruption, and altered water availability can increase exposure to diarrheal pathogens; ENSO relationships are location dependent (10, 11)	Water-quality surveillance, WASH preparedness, safe-water measures, and enhanced disease surveillance, adapted to local water infrastructure and flood vulnerability	Sanitation interventions can reduce diarrhoeal disease, although effect sizes vary. Direct evidence that ENSO-triggered timing improves effectiveness remains limited (18)
Altered vector ecology	ENSO-related temperature and precipitation variability can alter dengue risk, but effects differ considerably across countries and regions (7–9)	Entomological surveillance, source reduction, diagnostic readiness, and vector-control preparedness. Triggers should reflect local vector abundance, disease endemicity, seasonality, and historical climate–disease relationships	Some vector-control interventions have direct efficacy evidence; *Wolbachia*-based control reduced dengue in a cluster-randomized trial. Evidence for activating such measures specifically in response to ENSO forecasts remains limited (19)
Drought, food insecurity, and malnutrition	Drought can influence water security, agricultural production, food access, livelihoods, and nutrition through environmental and socioeconomic pathways (2–4)	Water-security planning, nutritional surveillance, food-security monitoring, and protection of essential supplies, tailored to local food systems and social vulnerability	Primarily supported by established preparedness and risk-management practice; comparative evidence for ENSO-triggered interventions remains limited
Population displacement and interruption of care	Flood, drought, and livelihood disruption can contribute to displacement, overcrowding, interrupted healthcare, and increased vulnerability (2–4)	Continuity of essential medicines and care, vaccination readiness, communicable-disease surveillance, and psychosocial support, adapted to local mobility patterns and service access	Supported mainly by emergency preparedness and operational guidance rather than trials of ENSO-triggered interventions (20)
Health-system disruption	Climate-related hazards can interrupt electricity, water, laboratories, cold chains, transport, communications, and routine care (2–4)	Backup power and water, protected cold chains, contingency stocks, surge readiness, and continuity-of-operations plans based on local facility vulnerabilities	Strongly supported as preparedness practice, but direct evidence comparing forecast-triggered with reactive activation remains sparse (20)

WASH, water, sanitation and hygiene.

These gaps should be stated openly rather than filled by assumption. Doing so does not weaken the case for anticipatory preparedness; it clarifies which actions are supported by intervention evidence, which are based primarily on established preparedness practice, and which require prospective evaluation.

## 5 Equity and one health as decision criteria, not add-ons

A technically accurate forecast can still produce a poor public health response if vulnerability and equity are considered only after decisions have been made.

Children may be particularly vulnerable to diarrheal disease, malnutrition, and disruption of essential services. Older adults and people with chronic disease are at greater risk during extreme heat. Pregnant women may face the combined effects of heat exposure, food insecurity, environmental disruption, and reduced access to maternal care. Outdoor workers may have little control over occupational exposure, while displaced and economically marginalized populations frequently experience several hazards simultaneously.

An operational climate-health assessment should therefore identify not only where a hazard may occur, but who is most likely to be harmed and who may be least able to act on a warning.

Equity should influence trigger design in at least three ways.

First, vulnerability can justify an earlier threshold for no-regret action. A moderate climate risk affecting a population with limited water access, weak healthcare infrastructure, or high baseline disease burden may warrant earlier preparedness than the same forecast in a more resilient setting.

Second, the feasibility of recommended protective behavior must be considered. Advising people to remain cool is of limited value when cooling is unaffordable or electricity unreliable. Recommending safe water cannot protect communities where safe water is unavailable.

Third, evaluation should examine the distribution of benefit, not only aggregate outcomes. An intervention that lowers average population risk but fails to reach remote, displaced, low-income, or otherwise underserved groups cannot be considered fully successful.

One Health perspectives complement this equity-sensitive approach because climate variability can affect human populations, livestock, wildlife, vectors, water, food systems, and ecosystems simultaneously. Integrating meteorological, environmental, entomological, animal-health, and human-health information may identify emerging risks earlier and provide a more complete basis for locally appropriate action than sector-specific surveillance alone (7, 13).

## 6 Discussion: from conceptual framework to real-world testing

The principal strength of anticipatory action is also its principal challenge: decisions must be made before outcomes are known with certainty.

A false-positive trigger occurs when action is initiated but the anticipated health impact does not materialize. Consequences may include unnecessary expenditure, diversion of personnel, opportunity costs, loss of confidence, and alert fatigue.

A false-negative trigger occurs when the threshold is not reached but a harmful event subsequently develops, potentially resulting in a lost opportunity to strengthen surveillance, protect vulnerable communities, prepare health facilities, or prevent illness.

The acceptable balance between these errors depends on the intervention. Reviewing laboratory preparedness or updating a clinical alert is inexpensive and useful beyond a particular event and can therefore tolerate more false alarms. Large-scale deployment of resources, health-service reconfiguration, or other costly interventions requires greater confidence.

Opportunity costs deserve similar attention. Staff, laboratory capacity, emergency funds, vector-control teams, and communication resources directed toward one anticipated threat may no longer be available for other priorities. Decisions should therefore consider not simply whether an action could help, but whether the expected benefit justifies its cost and the competing health priorities that may be displaced (12, 14, 15).

Uncertainty should also be communicated rather than concealed. A useful warning should explain what is expected, how confident the assessment is, who may be affected, what action is recommended, and when the decision will be reassessed. Tiered alerts can help limit alert fatigue while allowing interventions to escalate as evidence strengthens or de-escalate when risk falls.

### 6.1 Empirical validation remains the critical next step

The proposed framework is intentionally conceptual. It organizes existing climate, epidemiological, preparedness, and anticipatory-action principles into a decision pathway, but it has not yet been prospectively validated as a complete public health intervention.

This limitation should be explicit;

A major evidence gap persists between showing that a climate signal predicts or is associated with a health risk and demonstrating that acting on that information improves population health. Existing evidence often evaluates individual components—forecast performance, climate–disease associations, or intervention efficacy—rather than the complete sequence from seasonal warning to health outcome (12, 13).

The next phase should therefore be real-world testing.

Initial implementation could begin in sentinel jurisdictions where climate–health relationships are reasonably well characterized and surveillance systems are sufficiently mature. Rather than immediately testing resource-intensive interventions, early evaluations could focus on no-regret and targeted preparedness measures.

Pilot implementation should assess:

- Whether forecast information reaches health decision-makers with sufficient lead time;
- Whether locally calibrated thresholds accurately discriminate meaningful risk;
- Whether recommended actions are actually implemented;
- Rates of false-positive and false-negative triggers;
- Operational and opportunity costs;
- Acceptability to decision-makers and communities;
- Whether vulnerable populations are reached equitably;
- Effects on surveillance timeliness and health-system readiness; and
- Ultimately, whether morbidity, mortality, or disruption of essential services is reduced.

Prospective cohort evaluations, interrupted time-series analyses, stepped implementation across jurisdictions, and other pragmatic or quasi-experimental approaches may be more feasible than conventional randomized trials for many preparedness interventions.

Importantly, implementation should be iterative. Thresholds that perform poorly should be recalibrated, ineffective actions removed, and local experience incorporated into subsequent seasonal cycles.

The framework should therefore be viewed not as a fixed product but as a learning system.

### 6.2 A research agenda for forecast-informed public health

Three areas deserve particular attention.

First, stronger evidence is needed on the incremental value of climate-informed timing. An intervention may already be effective, but the relevant question is whether acting earlier because of a forecast provides additional benefit over routine or reactive delivery.

Second, studies should examine whether locally calibrated frameworks outperform generic thresholds. This is particularly important where climate teleconnections, epidemiology, and health-system capacity vary substantially over short geographic distances.

Third, equity must become an evaluation outcome. Forecast-informed preparedness should be judged not only by aggregate morbidity or mortality but also by whether protection reaches populations with the greatest exposure, vulnerability, and least capacity to adapt.

There may also be longer-term consequences of major El Niño events. Recent work has associated historical events with persistent changes in mortality trajectories and life expectancy, although these population-level findings require cautious interpretation and do not imply direct predictability of individual outcomes from ENSO forecasts (16). This reinforces the need for evaluation beyond the immediate emergency period.

The incremental contribution of this Perspective is therefore not another general call for El Niño preparedness. It is the proposal that the pathway between climate forecast and public health response should be explicit, locally calibrated, equity-sensitive, proportionate, and empirically evaluable.

El Niño forecasts provide valuable lead time, but lead time is not certainty. The appropriate public health response lies between two extremes: automatically activating interventions whenever El Niño is forecast and waiting until adverse health outcomes are already evident.

A more defensible approach is to use the forecast to begin a structured decision process that combines climate science with local epidemiology, vulnerability, health-system capacity, intervention effectiveness, equity, costs, and evolving surveillance information.

Ultimately, the value of climate-informed public health should not be measured by how many alerts are issued. It should be measured by whether decision-makers act earlier when action is justified, avoid unnecessary interventions when evidence remains weak, protect those at greatest risk, preserve essential services, learn from each forecast cycle, and reduce preventable harm.

An El Niño forecast should begin a locally informed public health decision process not automatically dictate its outcome.

## Data Availability

The original contributions presented in the study are included in the article/supplementary material, further inquiries can be directed to the corresponding author/s.
